# Risk factors of sepsis and prevalence of multidrug‐resistant organisms in pediatric cardiac surgery in tertiary care teaching rural hospital in India: A retrospective observational study

**DOI:** 10.1002/hsr2.1191

**Published:** 2023-04-15

**Authors:** Vishal V. Bhende, Sohilkhan R. Pathan, Tanishq S. Sharma, Amit Kumar, Hardil P. Majmudar, Vishal A. Patel

**Affiliations:** ^1^ Department of Pediatric Cardiac Surgery, Bhanubhai and Madhuben Patel Cardiac Centre, Shree Krishna Hospital Bhaikaka University Anand Gujarat India; ^2^ Clinical Research Co‐ordinator, Clinical Research Services, Bhanubhai and Madhuben Patel Cardiac Centre, Shree Krishna Hospital Bhaikaka University Anand Gujarat India; ^3^ Department of Community Medicine SAL Institute of Medical Sciences Ahmedabad Gujarat India; ^4^ Department of Pediatric Cardiac Intensive Care, Bhanubhai and Madhuben Patel Cardiac Centre, Shree Krishna Hospital Bhaikaka University Anand Gujarat India

**Keywords:** antibacterial resistant, multidrug‐resistant infection, pediatric cardiac surgery, sepsis

## Abstract

**Background and aims:**

Cardiac surgery and cardiopulmonary bypass result in an immunoparalyzed state in children making them susceptible to sepsis and other hospital‐acquired infections. Therefore, identification of the risk factors of sepsis would lead to appropriate management. The current study seeks to evaluate the prevalence of sepsis and risk factors linked to sepsis in pediatric cardiac surgical patients and the subsequent prevalence of multidrug‐resistant organisms.

**Methods:**

A retrospective, single‐center observational study was conducted including 100 pediatric patients admitted to the pediatric intensive care unit (ICU) after cardiac surgery between January 2017 and February 2018. All patient data were obtained from the medical record department of the hospital. Patient case report form comprised demography, surgery details, preoperative and postoperative hematological reports, and clinical details. After collecting the data, chi‐square test and logistic regression analysis were used to determine the risk factors linked to sepsis.

**Results:**

The prevalence of sepsis in our population was 27% and the mortality rate due to sepsis was 1%. The only statistically significant risk factor for sepsis we discovered in this analysis was prolonged ICU stay for more than 5 days. A total of eight patients had blood cultures positive for bacterial infection. The alarming finding was that all eight were infected with multidrug‐resistant organisms, demanding the last line of antibacterials.

**Conclusion:**

Our study indicates that special clinical care is required when ICU stay is prolonged to lower the risk of sepsis. These new and upcoming infections not only promote high mortality and morbidity rates but also contribute to increased cost of care due to the use of newer broad‐spectrum antibiotics and longer hospital stay. The high prevalence of multidrug‐resistant organisms is unacceptable in the current scenario and hospital infection and prevention control play a crucial role in minimizing such infections.

## 1 INTRODUCTION

Improved surgical methods, cardiopulmonary bypass (CPB), and postoperative care have considerably increased the survival rate for neonates, infants, and children with congenital heart disease (CHD). The stimulation of the complement cascade, the release of endotoxin, the activation of leukocytes, and the production of pro‐inflammatory cytokines are all elements of the complicated, systemic inflammatory response that is influenced by CPB. This complex cellular and humoral immune response results in a relative and transient state of immune suppression, often called “immunoparalysis.”^1^, ^2^ This state of immunoparalysis may result in an increased risk of sepsis in children undergoing cardiac surgery.^3^, ^4^ Furthermore, chronic hypoxia, low cardiac output syndrome, and other comorbid conditions linked to CHD,^3^, ^4^, ^5^ as well as invasive devices may also elevate the risk of sepsis in this population.

Despite extensive adoption of healthcare guidelines and sepsis prevention strategies, the frequency of healthcare‐acquired infection remains high, at 6%–30.8% in pediatric cardiac surgical patients. Sepsis is a significant and individual risk factor for increased duration of mechanical ventilation, cardiac intensive care unit (ICU) length of stay, healthcare costs, and mortality in children with CHD.

Very few case series of sepsis prevalence in pediatric cardiac surgical patients, in a rural tertiary care hospital from India have been documented. None of them has indicated the lingering threat of increasing prevalence of multidrug‐resistant organisms.

In this study, we aimed to assess the prevalence of sepsis and the risk factors associated with it in pediatric cardiac surgical patients and the subsequent prevalence of multidrug‐resistant organisms.

## 2 MATERIALS AND METHODS

### 2.1 Study design and setting

This was a retrospective, single‐center observational study undertaken at the Cardiac Paediatric ICU (CPICU) of Bhanubhai and Madhuben Patel Cardiac Centre, Shree Krishna Hospital, Karamsad, India after getting the authorization for the research project from the Institutional Ethics Committee (IEC‐2) of the HM Patel Centre for Medical Care and Education, Anand, Gujarat, India, vide approval No. (IEC/BU/2021/Ex.03/44/2023 dated 24.01.2023). The study is carried out following the Helsinki standards, 2013 and STROBE standards of observational studies.

### 2.2 Inclusion criteria

- Consecutive Paediatric Patients less than or equal to 14 years old were admitted to the CPICU after pediatric cardiac surgery between January 2017 and February 2018.

### 2.3 Exclusion criteria

- Older patients and patients undertaking any major non‐cardiac surgical intervention.
- Also, patients discharged against medical advice, having a preadmission infection, or patients transferred from other hospital departments or other centers with infection.

### 2.4 Data collection

The study was implemented after receiving authorization from the Institutional Ethics Committee of the hospital. It was a retrospective observational study so the Informed consent form was rescinded by the Institutional Ethics Committee.

The study included 100 pediatric patients (less than 14 years) who had cardiac surgery between January 2017 and February 2018. All the data of the patients were received from the medical record department of the hospital. The case report form was compiled in English.

To look for the related risk factors for sepsis patients clinical and laboratory data were obtained in case report form. Such data are the patient's demographic data including sex, age, weight, height, cardiac and noncardiac abnormalities, and major medical history. Preoperative sepsis screen and risk factors of immunesuppression like severe mal‐nutrition, chromosomal disorders, HIV infection, and use of preoperative antibiotics and hospital stay were obtained.

Surgery data were obtained with surgery type, risk adjustment for congenital heart surgery (RACHS) score for assessing surgical complexity, CPB duration, hypothermia or total circulatory arrest, delayed sternal closure, intubation and ventilation duration, need for reintubation, central catheter lines, and its duration, length of ICU stay, blood transfusion, inotropic support, renal support, and tracheostomy.

Hematology data included were preoperative and postoperative hemoglobin level, leukocytes count, C‐reactive protein, procalcitonin, and culture reports recorded from the medical records of the patients. In the case of a positive culture report, antibiotics sensitivity sequence and their minimum inhibitory concentration were recorded. Mortality with its cause was also highlighted.

The definition of sepsis and septic shock was determined as per the definition mentioned in Sepsis‐3. Suspected sepsis was labeled when a patient was managed as sepsis, based on clinical and laboratory parameters.

### 2.5 Statistical analysis

Data were expressed as mean and standard deviation with minimum and maximum values or as numbers and percentages according to the type of data. An Independent student *t*‐test was employed for the comparison of numerical data and a chi‐square test for categorical data. Logistic regression analysis was employed to evaluate the risk factors linked to sepsis.

## 3 RESULTS

One hundred and three pediatric patients with age less than 14 years, had undertaken cardiac surgery during the period from January 2017 to February 2018. Three patients who were discharged against medical advice or transferred to other centers were exempted from the study.

One hundred patients, 55 males and 45 females, were eventually included in this study and were observed retrospectively for the occurrence of sepsis after cardiac surgery (Table 1).

**Table 1:** Demographic and surgical characteristics.

Characteristics	Mean (SD)	SD	Median	Minimum	Maximum
Age (year)	2.39	3.29	0.9	1 (day)	14 (year)
Weight (kg)	8.14	5.72	5.93	1	28
Height (cm)	78.02	25.34	70	43	144
Risk adjustment for congenital heart surgery (RACHS)	1.85	0.74	2	1	4
Surgery duration (min)	89.43	39.69	83	28	230
Duration of intubation (h)	40.62	51.07	24	1	336
Preoperative length of stay (days)	1.93	3.10	1	0	28
Total no. of hospital stays (days)	9.58	5.61	8	3	35
Length of ICU stay (days)	3.91	4.46	2	0	26

Regarding patients' weight, 58 patients were more than 5 kg and 36 patients were between 2.5 and 5 kg, whereas only six patients were less than 2.5 kg in weight.

A total of eight patients were in the age group of <30 days (neonates), 44 patients were in the age group of 30 days–1 year (infants), 30 patients were in the age group of 1–5 years, and 18 patients were in the age group of 5–14 years.

In this research, eight patients had blood culture‐proven sepsis. Another eight patients had suspected sepsis that emerged after surgery, while 11 had suspected sepsis that emerged preoperatively during hospital stay (Table 2).

**Table 2:** Infection and mortality.

	*N* = 100	%
Proven sepsis	8	8
Suspected sepsis	19	19
No infection	73	73
Mortality	‐	2

In our study group, there was no report of mediastinitis or endocarditis. Although six patients had sternal wound gaping. Swab culture from these patients was sterile. They improved on oral antibiotic administration. Two patients died (Table 3).

**Table 3:** Mortality in the study group.

Mortality	Sepsis	Risk adjustment for congenital heart surgery (RACHS)	Cause of death
Patient 1	Yes	4	Acute respiratory distress syndrome with Sepsis
Patient 2	No	3	Cardiogenic shock

Among the type of surgery, ventricular septal defect (VSD) closure surgery had a greater prevalence. A total of 14 patients underwent VSD closure, and 4 (28%) of them had sepsis.

As we inserted risk factors data in chi‐square statistics (Table 4), we got seven significant *p*‐values which were <0.05. The significant risk factors include age (0.005), weight (0.004), surgery duration (0.000), length of ICU stay (0.000), duration of intubation (0.001), duration of central venous catheterization (0.001), and delayed sternal closure (0.001).

**Table 4:** Clinical characteristics of the patient population and risk factors for sepsis in bivariate analysis (chi‐square statistics).

Variable	Level	Total (*N* = 100)	Overall %	Number with sepsis (*n* = 27)	% with sepsis	Number with no sepsis (*n* = 73)	% with no sepsis	*p‐*Value
Preoperative assessment								
RACHS category	1	34	34	5	18.52	29	39.73	0.236
	2	49	49	16	59.26	33	45.21	
	3	15	15	5	18.52	10	13.70	
	4	2	2	1	3.70	1	1.37	
	5	0	0	0	0	0	0	
	6	0	0	0	0	0	0	
Demographics								
Age	<30 days	8	8	5	18.52	3	4.11	**0.005**
	30 days–1 year	44	44	16	59.26	28	38.26	
	1–5 year	30	30	5	18.52	25	34.25	
	5–14 year	18	18	1	3.70	17	23.29	
Weight	<2.5 kg	6	6	4	14.81	2	2.74	**0.004**
	2.5–5 kg	36	36	14	51.85	22	30.14	
	>5 kg	58	58	9	33.33	49	67.12	
Sex	Male	55	55	15	55.56	40	54.79	0.946
	Female	45	45	12	44.44	33	45.21	
Risk factors								
Preoperative length of stay (days)	0	9	9	5	18.52	4	5.48	0.156
	1	60	60	13	48.15	47	64.38	
	2	14	14	5	18.52	9	12.33	
	>3	17	17	4	14.81	13	17.81	
Surgery duration (min)	<30	1	1	1	3.70	0	0	**0.000**
	30–90	62	62	8	29.63	54	73.97	
	90–200	35	35	18	66.67	17	23.29	
	>200	2	2	0	0	2	2.74	
Length of ICU stay (days)	1	34	34	1	3.70	33	45.83	**0.000**
	2–4	37	37	9	33.33	28	38.89	
	5–9	19	19	10	37.04	9	12.50	
	10–30	9	9	7	25.93	2	2.78	
Duration of intubation (h)	3	21	21	1	3.70	20	27.40	**0.001**
	8	9	9	1	3.70	8	10.96	
	9–120	64	64	20	74.07	44	60.27	
	>120	6	6	5	18.52	1	1.37	
Duration of central venous catheterization (h)	3	21	21	1	3.70	20	27.40	**0.001**
	8	9	9	1	3.70	8	10.96	
	9–120	64	64	20	74.07	44	60.27	
	>120	6	6	5	18.52	1	1.37	
Reintubation	Yes	10	10	4	14.21	6	8.22	0.329
	No	90	90	23	85.19	67	91.78	
Inotropic Support	Yes	63	63	21	77.78	42	58.33	0.073
	No	36	36	6	22.22	30	41.67	
Delayed sternal closure	Yes	7	7	6	22.22	1	1.37	**0.001**
	No	93	93	21	77.78	72	98.63	
Hypothermia	Yes	47	47	17	62.96	30	41.10	0.052
	No	53	53	10	37.04	43	58.90	

*Note*: Bold values are statistically significant.

Multivariate logistic regression analysis was conducted to evaluate the risk factors of sepsis (Table 5). The analysis revealed that the risk factors were linked only to the length of ICU stay. Prolonged duration of ICU stay of more than 5 days (<0.05) was a statistically significant risk factor for sepsis.

**Table 5:** Risk factors for sepsis in multivariable analysis (logistic regression analysis).

Variable	Level	Number with sepsis (*n* = 27)	% with sepsis	Number with no sepsis (*n* = 73)	% with no sepsis	Odds ratio (OR)	(95% confidence interval [CI])	*p*‐Value
Age	<30 days–1 year	16	59.26	28	38.26	1.18	[0.14, 9.70]	0.87
	1–5 year	5	18.52	25	34.25	0.82	[0.05, 12.5]	0.89
	5–14 year	1	3.70	17	23.29	0.40	[0.01, 11.3]	0.59
Weight	2.5–5 kg	14	51.85	22	30.14	0.66	[0.07, 5.5]	0.70
	>5 kg	9	33.33	49	67.12	0.68	[0.06, 7.3]	0.75
Length of ICU stay (days)	2–4	9	33.33	28	38.89	9.03	[0.82, 98.8]	0.07
	5–9	10	37.04	9	12.50	19.0	[1.5, 226.8]	**0.02**
	10–30	7	25.93	2	2.78	50.4	[2.56, 994.0]	**0.01**
Duration of intubation (h)	8	1	3.70	8	10.96	1.24	[0.05, 28.9]	0.89
	9–120	20	74.07	44	60.27	4.45	[0.46, 42.9]	0.19
	>120	5	18.52	1	1.37	9.52	[0.29, 311.0]	0.20
Delayed sternal closure	Yes	17	62.96	30	41.10	0.36	[0.003, 43.0]	0.68
	No	10	37.04	43	58.90	0.3	[0.001, 1.0]	0.054

*Note*: Bold values are statistically significant.

The eight patients who had blood cultures positive for bacterial infection mostly were positive for nosocomial gram‐negative bacteria. One was *Enterococcus faecium*. The unsettling discovery is that except one, all others were multidrug‐resistant organisms requiring the last line of antibacterials as displayed in the chart.

## 4 DISCUSSION

Infections in children are frequent (incidence, 13%–31%) after cardiac surgery.^3^, ^6^ Many are surgical site infections (incidence, 2.3%–8%).^7^ Some are more severe, such as septicemia (incidence, 6.3%–15%),^8^, ^9^, ^10^ mediastinitis (incidence, 0.2%–3.3%),^6^, ^7^, ^8^, ^9^, ^10^, ^11^, ^12^, ^13^, ^14^ and endocarditis (incidence, 0.2%).^11^

Pollock et al.^4^ reported blood‐stream infections (BSIs) in 21 (6.8%) out of 310 children after cardiac surgery. In our research, the prevalence of sepsis was 27% with a 1% mortality rate. There were no cases of mediastinitis or endocarditis in the studied group. A total of 6% of patients had surgical site infections.

Fakhri et al.^12^ indicated in their study that the duration of CPB ≥ 90 min. was linked to a 5.538 increased risk of postsurgical sepsis in comparison to those ≤90 min. Our study was inadequately powered to verify it, although trends indicated it.

Rosanova et al.^13^ concluded that longer hospitalization and inotropic support were risk factors for infections in their analysis.

Hatachi et al.^14^ concluded that mechanical ventilation greater than or equal to 3 days, dopamine use, genetic abnormality, and delayed sternal closure were linked to healthcare‐associated infections after pediatric cardiac surgery.

Longer duration of ICU stay (more than 5 days) was a significant risk factor for sepsis in our research. Multivariate analysis did not reveal other independent risk factors. This may be because of insufficient data to achieve statistical significance.

However, the unsettling discovery in our study is the very high incidence of multidrug‐resistant nosocomial bacterial infection (seven of eight patients with culture‐positive sepsis). They required the last line of antibacterials. Despite this, the mortality rate influenced by sepsis was only 1%. However, mortality could be exacerbated if we lost the sensitivity to the last line of antibacterials. This scenario is intolerable in pediatric cardiac patients who may experience precarious hemodynamics due to cardiac surgery.

Other studies have also outlined the prevalence of resistant organisms in pediatric cardiac septic patients. This demonstrates deficiencies in antisepsis measures in many pediatric cardiac departments and the need for a highly officious infection control strategy.

## 5 CONCLUSION

The prevalence of sepsis in this research was 27%, which is similar to other studies. With the application of adequate sepsis guidelines, its prevalence can be decreased further to 5% as in a recent study.

There is a high incidence of multidrug‐resistant organisms in culture‐positive patients with sepsis. If immediate steps are not taken to contain it, we may lose many patients because of sepsis caused by resistant bacteria. Misuse and overuse of antibiotics may further worsen the problem.

In this study, prolonged ICU stay was a statistically significant risk factor for sepsis. It indicates that special clinical care is required when ICU stay is prolonged to lower the risk of sepsis.

## 6 LIMITATIONS OF THE STUDY

As it is a retrospective study, some data discrepancies cannot be exempted. Although care has been taken to minimize it. Many other risk factors were not statistically significant because the study group was not sufficiently powered. As it is a single‐center study, its findings cannot be generalized for entire pediatric cardiac surgery centers in a region or country.

## AUTHOR CONTRIBUTIONS

**Vishal V. Bhende**: Conceptualization; data curation; formal analysis; funding acquisition; investigation; methodology; project administration; resources; software; supervision; validation; visualization; writing—original draft; writing—review and editing. **Sohilkhan R. Pathan**: Data curation; formal analysis; investigation; methodology; project administration; resources; software; supervision; validation; visualization. **Tanishq S. Sharma**: Data curation; formal analysis; funding acquisition; investigation; methodology; project administration; resources; software; supervision; validation; visualization; writing—original draft; writing—review and editing. **Amit Kumar**: Data curation; formal analysis; investigation; methodology; project administration; software; supervision; validation; visualization. **Hardil P. Majmudar**: Data curation; formal analysis; investigation; methodology; project administration; resources; software; supervision; validation; visualization. **Vishal A. Patel**: Data curation; formal analysis; project administration; resources; supervision; validation.

## CONFLICT OF INTEREST STATEMENT

The authors declare no conflict of interest.

## TRANSPARENCY STATEMENT

The lead author Vishal V. Bhende affirms that this manuscript is an honest, accurate, and transparent account of the study being reported; that no important aspects of the study have been omitted; and that any discrepancies from the study as planned (and, if relevant, registered) have been explained.

## Data Availability

The data that support the findings of this study are openly available in doi:10.22541/au.167543647.76773927/v1.
